# Development of a training programme for primary care providers to counsel patients with risky lifestyle behaviours in South Africa

**DOI:** 10.4102/phcfm.v7i1.819

**Published:** 2015-06-05

**Authors:** Zelra Malan, Bob Mash, Katherine Everett-Murphy

**Affiliations:** 1 Family Medicine and Primary Care, Stellenbosch University, South Africa; 2 Chronic Diseases Initiative in Africa (CDIA), Faculty of Health Sciences, University of Cape Town, South Africa

## Abstract

**Background:**

We are facing a global epidemic of non-communicable disease (NCDs), which has been linked with four risky lifestyle behaviours. It is recommended that primary care providers (PCPs) provide individual brief behaviour change counselling (BBCC) as part of everyday primary care, however currently training is required to build capacity. Local training programmes are not sufficient to achieve competence.

**Aim:**

This study aimed to redesign the current training for PCPs in South Africa, around a new model for BBCC that would offer a standardised approach to addressing patients’ risky lifestyle behaviours.

**Setting:**

The study population included clinical nurse practitioners and primary care doctors in the Western Cape Province.

**Methods:**

The analyse, design, develop, implement and evaluate (ADDIE) model provided a systematic approach to the analysis of learning needs, the design and development of the training programme, its implementation and initial evaluation.

**Results:**

This study designed a new training programme for PCPs in BBCC, which was based on a conceptual model that combined the 5As (ask, alert, assess, assist and arrange) with a guiding style derived from motivational interviewing. The programme was developed as an eight-hour training programme that combined theory, modelling and simulated practice with feedback, for either clinical nurse practitioners or primary care doctors.

**Conclusion:**

This was the first attempt at developing and implementing a best practice BBCC training programme in our context, targeting a variety of PCPs, and addressing different risk factors.

## Abstrait

**Développement d’un programme de formation pour prestataires de soins primaires pour leur permettre de conseiller les patients à comportement à risque en Afrique du Sud**.

**Contexte:**

Nous sommes confrontés à une épidémie globale de maladies non transmissibles (MNT), liées à quatre comportements à risque. Nous recommandons que les prestataires de soins primaires (PSP) offrent des consultations individuelles visant à modifier les comportements (CIMC) dans le cadre des soins de santé primaire; cependant, il faudra organiser des formations pour renforcer les capacités. Les programmes de formation locaux ne suffisent pas pour développer les compétences.

**Objectif:**

Cette étude avait pour but de remanier la formation actuelle des PSP en Afrique du Sud, autour d’un nouveau modèle pour la CIMC qui offrirait une approche normalisée pour s’occuper des comportements à risque des patients.

**Lieu:**

La population de l’étude comprenait des infirmières cliniques et des médecins de soins primaires dans la province du Western Cape.

**Méthodes:**

Le modèle ADDIE a offert une approche systématique à l’analyse des besoins d’apprentissage, à la conception et au développement du programme de formation, à sa mise en œuvre et à son évaluation initiale

**Résultats:**

Cette étude a conçu un nouveau programme de formation pour les PSP en CIMC, basé sur un modèle conceptuel combinant 5 processus (demander, alerter, évaluer, assister et organiser) avec un style directeur fondé sur l’entrevue motivationnelle. Le programme a été conçu comme un programme de formation de huit heures combinant la théorie, la modélisation et la simulation avec des feedback, pour les infirmières cliniciennes ou les médecins de soins primaires.

**Conclusion:**

C’était la première fois qu’on élaborait et mettait en œuvre un programme de formation de meilleure pratique en CIMC dans notre contexte, qui ciblait une variété de PSP, et abordait les différents facteurs de risques.

## Introduction

Many countries, including South Africa, are reporting an increase in the prevalence of non-communicable diseases (NCDs) such as hypertension and diabetes. These NCDs have been linked to underlying risky lifestyle behaviours such as tobacco smoking, unhealthy diet, alcohol abuse and physical inactivity.^1,2^ Brief behaviour change counselling (BBCC), which is integrated into routine primary care, can be effective in helping patients change these risky behaviours.^3,4,5^ The importance of this has been recognised by the South African Department of Health in its National Strategic Plan for the Prevention and Control of NCDs.^6^ Capacitating primary care providers (PCPs) to deliver BBCC in primary care settings is recommended for all four risk factors.

It is feasible for BBCC to be effectively delivered by PCPs, who have both the opportunity and credibility to do so.^3,4,5^ Training can enhance PCPs efficiency and capacity to provide this counselling, provided adequate resources and support are available in the workplace.^5,7^ Most of the research that explores training interventions for PCPs to deliver this counselling is from developed countries, such as the USA, Australia and Canada.^7,8,9^ The few studies that have been undertaken locally suggest that such training is currently inadequate.^10,11,12^

PCPs face a difficult task in helping patients to change their risky lifestyle behaviours. In South Africa the experience of counselling in practice tends to be discouraging, because of the poor response of patients, and challenging because of numerous barriers. These include language barriers, lack of time, poor knowledge of lifestyle modification, inadequate counselling skills, lack of self-efficacy, and poor continuity of care. PCPs reported lack of confidence in their ability to help patients change and their scepticism about the effectiveness of lifestyle counselling may partly be a reflection of inadequate training in this area.^10,11^

Recent situational analyses in the Western Cape showed that current training programmes on behaviour change counselling for PCPs are not sufficient to achieve competence in clinical practice.^10,11^ Training programmes were mostly theoretical and without the opportunity to practise key skills, and receive constructive feedback on performance. PCPs therefore were not confident to perform BBCC. Due to time constraints, training was not integrated throughout the curriculum and there was no reinforcement afterwards. The training outcomes were not assessed, and even lecturers were unaware of the evidence in support of BBCC and had low confidence in the effectiveness of their training.^11^ Therefore trainers, and not just students, needed training in BBCC.

Traditionally PCPs rely mostly on the directive style when counselling patients on behaviour change, resulting in resistance from the patient, and frustration for the PCPs.^13^ Changing the PCPs current style of communication could be challenging, and we realised that the training should focus not only on teaching PCPs a structure for BBCC and evidence-based knowledge of lifestyle modification, but also to transform their style of communication.

Capacitating PCPs to deliver BBCC in a skilful way that is integrated within the consultation is essential.^3,4,14^ This study aimed to address this need by redesigning the current training around a new model for BBCC that would offer a standardised approach to addressing different NCD risk behaviours, be realistic in terms of training time and resources, be feasible to perform in clinical practice, as well as evidence-based and effective.

## Aims and objectives

The aim of this study was to develop a training programme for PCPs that delivered a best practice BBCC method for patients with risky lifestyle behaviours and evidence-based information on NCDs. This paper focuses on the development of the training intervention; the evaluation of it in clinical practice will be reported on at a later date. Specific objectives were to:

- Design a best practice BBCC training programme to meet the needs of PCPs.
- Develop the structure and content of the training intervention, as well as the skills and resources needed to deliver this programme.
- Implement the training programme.
- Evaluate and revise the training programme.

## Research methods and design

### Study design

**FIGURE 1:**
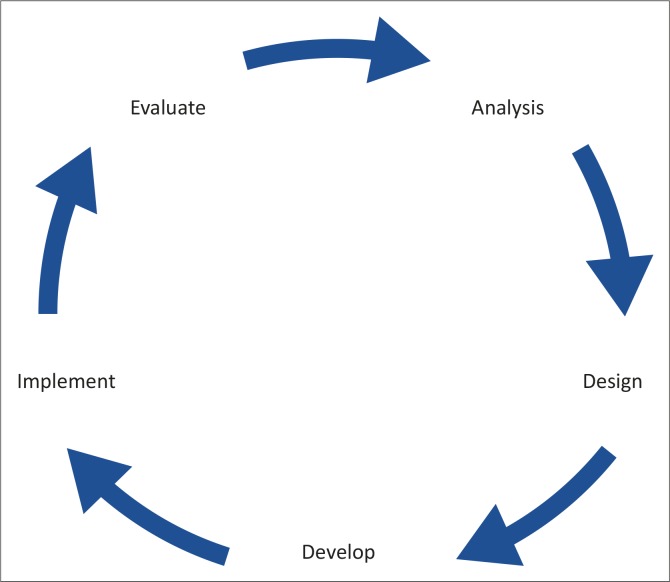
The ADDIE model for design of training programmes.

Most approaches to instructional systems design reflect the ADDIE (analyse, design, develop, implement and evaluate) model, which uses a systemic problem solving approach to develop new training programmes.^15^ The ADDIE model may be particularly useful if the main focus of the new training programme is changing the behaviour of participants.^15^ The objectives above were based on the ADDIE model, which is also shown in Figure 1. The ADDIE model provided a systematic approach to the analysis of learning needs, the design and development of the training programme, its implementation and initial evaluation.^15^ The ADDIE process had also been previously used in local educational research and development of training programmes.^16,17^ The methods used to complete each step of the model are described below.

### Setting

Eighty percent of the South African population make use of public sector health care facilities, particularly those with NCDs, which are amongst the commonest conditions seen in primary care.^18^ The majority of patients are seen by clinical nurse practitioners in either small clinics or larger health centres. Clinical nurse practitioners only receive an additional one year of training to cope with the wide range of problems seen in primary care. Clinical nurse practitioners are supported by primary care doctors, who usually have no additional postgraduate training. Therefore, the PCPs targeted by this training programme were clinical nurse practitioners and primary care doctors.

The training programme was developed with the intention of initially implementing and evaluating it as part of the one-year Diploma course (Diploma in Clinical Nursing Science, Health Assessment, Treatment and Care) at Stellenbosch University, which focused on training clinical nurse practitioners, and the four-year postgraduate training programme in family medicine, which was aimed at doctors, at both the University of Cape Town and Stellenbosch.

### Educational team

An educational team was created to collaborate on the ADDIE process and consisted of the three authors. The team combined expertise in motivational interviewing, adult education, NCDs, primary care research and brief behaviour change counselling (BBCC).^16,17^^18,19,20,21,22^

### ADDIE process

#### Step 1 Analysis

The situational analysis of current training and clinical practice for the target audience has been summarised in the introduction and published elsewhere.^11^ This article reports on the remaining ADDIE process.

#### Step 2 Design

In the design stage the educational team developed a conceptual model and learning outcomes that were derived from the findings of the situational analysis and a literature review. The literature review searched PubMed, Google Scholar and local African journals for evidence on effective models of BBCC, as well as approaches to the design and implementation of training programmes.

#### Step 3 Development

The educational team then developed the structure and content of the training programme, learning activities and strategies, as well as the educational resources required. Care was taken to ensure that all of these elements were aligned with the design of the learning outcomes and conceptual model.

The educational team then prepared the actual content, for instance power point slides, instructions for role plays and practical exercises, by drawing on existing materials and previous experiences in training motivational interviewing. This process also helped the team to determine which resources were available and which needed to be developed, or obtained.

The training programme that emerged from the design and development phases was presented to a group of programme managers, clinicians and trainers of family medicine and primary care at Stellenbosch University to obtain further input from experts in primary health care.

#### Step 4 Implementation

During 2012–2013 three groups of nurses and three groups of primary care doctors completed the training programme at Stellenbosch University.

#### Step 5 Evaluation

Feedback forms from the training courses during 2012–2013 were used to obtain the participants’ opinions on how to improve the model, content or approach to teaching. The trainers analysed the information, and used it to revise the training programme accordingly. Revisions were also made based on the reflections of the educational team on their training experience.

## Ethical considerations

This study was approved by the Health Research Ethics Committees (HREC) at Stellenbosch University (Reference number: N11/11/321), on 27 February 2012.

## Results

### Design

The Canadian Task Force on Preventative Health, the Royal Australian College of General Practitioners, the National Health Service in the UK, the Department of Health and Aging in the Australian government and the Kinect consortium, all recommend the use of the 5 A construct in BBCC and promote its integration into primary care.^3,4,7,8^ The 5A construct consists of five steps: ask, advise, assess, assist and arrange. It provides PCPs with a broad framework for BBCC, which is simple to understand, applicable to different settings, and can be used by PCPs from different disciplines and levels of expertise.

Motivational interviewing (MI) is a flexible, evidence-based, clinical skill that can be used when talking to patients about how and why they might change their behaviour.^13,23^MI provides an alternative approach to the usual more authoritarian approaches and outperforms traditional advice-giving in 80% of studies.^22^ PCPs, who have little time with patients, can expect a 10% – 15% improvement in patients changing their risky lifestyle behaviours after counselling based on motivational interviewing (MI).^23^ At the core of MI lies the guiding style, which promotes collaboration, empathy, evocation, respect for choice and control, and a clear focus.

A guiding style communicates an approach of ‘I can help you solve this for yourself’ as opposed to ‘This is what you must do’. To accomplish this, PCPs need to be able to switch from their traditional role as directive, expert advice givers, to guides that can skilfully assist the patient to make difficult decisions about change.

Training practitioners to be competent in MI is not easy, and in our context unlikely to be achievable with the majority of PCPs in a short training course.^19,24^The decision was taken therefore to rather focus on the characteristics of a guiding style derived from MI and the essential underlying communication skills.^19,22,25^ The recommended approaches to completing each of the steps in the 5A model were rewritten to adopt this guiding style. The title of the second step, which was ‘advise’ in the original model, was changed to ‘alert’ as unsolicited advice-giving is seen as incongruent with a guiding style. The concept of alerting someone to a potential risk and evoking their response was, however, seen as more congruent with a guiding style.

The underlying spirit of the guiding style was based on three elements, which were collaboration vs. confrontation between the provider and the patient, evoking or drawing out the patient's ideas about change rather than imposing ideas, and emphasising the patient's autonomy versus being authoritative.^13,14,22^ Core key communication skills that support the guiding style were defined as asking open-ended questions, reflective listening, affirmations and summaries (OARS).^13^ Reflective listening and summarisation are perhaps the most crucial skills, which demonstrate empathy and encourage the patients to elaborate, whilst the use of open-ended questions invites patients to consider how and why they might change. Offering affirmations helps the patient to recognise their own strengths.

Therefore, in our model of BBCC the key principles of a guiding style were integrated into each step of the 5As structure as shown in Table 1. This approach to BBCC was supported by a local quasi-experimental study that demonstrated its effectiveness in helping pregnant women reduce, or stop, tobacco smoking when delivered by nurse midwives and lay counsellors.^20^

**TABLE 1:** Model of brief behaviour change counselling.

Step in the 5 As	Tasks in a guiding style
Ask	Identify risk behaviour and document in record. Ask the patient what he/she already knows about the risks associated with the behaviour or would like to know. Respectfully affirm what he/she knows Request permission to provide further information.
Alert	Provide relevant information in a neutral manner: Before giving information, emphasise that your role is to assist the patient in making informed choices, not to compel them to a particular course of action. Offer information on the health risks or benefits of change in a neutral way. Provide information using the ‘E-P-E’ method which is to elicit what the patient already knows and wants to know, provide relevant information, and elicit the patients understanding of this. If there is already a health problem related to the risk behaviour, clearly link the two.
Assess	Assess readiness to change: Ask the patient how they feel about the information provided and the possibility of making a change at this time. Assess how important change is for the patient and how confident he/she feels about change. Recognise and respond to ‘change talk’, which are statements by the patient revealing a desire, ability, reason, need or commitment to change. Offer support and assistance, but respect the patient's decision.
Assist	Offer help if he/she comes to a decision to change in the future. Request permission to give patient materials (if available), which could assist in them making a decision in the future. If response is ‘Yes, am ready to change’ then provide practical assistance to change such as: Positively reinforce any intentions to change which the patient expressed, no matter how small they may be. Express confidence in their capacity to achieve their health goal. Offer materials which teach behavioural change strategies and skills and express confidence that they will help. Prompt the patient to anticipate problems and barriers and to consider how to overcome these. Prompt patient to seek social support in their social environment. Prescribe medication, if appropriate.
Arrange	Arrange for follow up and/or referral: Document decisions made and materials given in the clinic record, add a reminder to discuss progress during the next visit and schedule follow up contact. Reiterate your and clinic staff's commitment to provide further information and support during behavioural change process. Refer patient to other health care providers for more intensive counselling if possible or to community based resources.

Based on this conceptual model, the results of the situational analysis and learning outcomes identified from the literature, as well as the educational team's own expertise in MI, the following learning outcomes were developed. At the end of the training programme participants would be able to:

1. Use a guiding style of counselling.
2. Practice reflective listening.
3. Recognise, elicit, and respond to change talk.
4. Exchange information.
5. Assess readiness to change.
6. Use the 5A steps (Ask, Alert, Assess, Assist, Arrange).
7. Counsel patients regarding the four key risk factors.

### Development

A key principle of this stage was to develop an educational process that itself resonated with the guiding style. In essence we recognised that we were asking PCPs to change their own behaviour regarding how they counselled other people about behaviour change. Therefore, the principles being recommended for BBCC should also be embodied in the course itself regarding its structure and process.

These principles needed not only to be embodied in the structure and process of the course, but also modelled by the trainers themselves. In other words, the trainer's style of training needed to be congruent with the style of counselling being taught.^26^

Behaviourist learning theory is particularly useful when a change in behaviour is the desired outcome of a training intervention.^27^ This approach recognises three key areas: namely, that the desired behaviour is the clear focus of the learning, that participants can practice the behaviour in a controlled and safe environment and that the training reinforces the behaviour by providing feedback on performance. Typically, the teacher demonstrates the specific behaviours, whilst participants observe, and then they practice and receive feedback on their own performance under controlled circumstances.^27^

The training was designed to give participants time to create a safe, supportive and reflective group environment.^26^ Group discussions provide an essential component of training, and to ensure participation from every participant, group size should be limited to 20 or less. For this training programme, a ratio of one trainer to 12 students was thought to be possible, but ideally one trainer to eight participants was seen as the ideal.

Traditional didactic teaching methods were not considered effective in changing behaviour.^26^ Although the training included some brief sharing of theory using didactic methods, these were combined with other approaches, such as the jig-saw method, in which members of a small group collectively master a piece of the whole theory and then share this piece with others in a new small group that includes peer-experts in all the other pieces. After information was given in a didactic manner the participants were also encouraged to reflect as a group, or individually, and consider their response to the theory or ideas presented.^26^ The programme included information on the specific risk factors, as this improves participant content knowledge and confidence in delivering effective interventions.^26^^,28^

Based on best evidence from the literature review, the following principles were used in the development of the training course:^26,28,29^

- Provide evidence of the current deficiencies in counselling, the reasons for them, and the consequences for patients and doctors.
- Offer an evidence base for the skills needed to overcome these deficiencies.
- Demonstrate the skills to be learned.
- Provide the opportunity to practice these skills.
- Give constructive feedback on performance.

Based on the time that could reasonably be made available for the training within the selected curricula for nurses and doctors, the training was designed as an eight-hour workshop, with four two-hour sessions. The final training programme that was developed is summarised in Table 2.

**TABLE 2:** Summary of the training programme.

Session	Time (minutes)	Purpose of session	Activities for session
1.1	15	Introductions and overview of programme and learning outcomes	Introduce the training programme in terms of the people involved, the intended learning outcomes and the process to be followed.
1.2	30	Understand participant's own prior experience of the challenges and successes of BBCC	Invite students to reflect in pairs and then share with the whole group on their prior experience with BBCC. This step was thought to be important in terms of building rapport between the trainers and participants, understanding the participant's context, allowing them to express their ambivalence and frustrations and have these recognised, and helping to focus attention on behaviour change counselling.
1.3	45	Evidence for BBCC	Provide evidence of the current deficiencies in counselling, the reasons for them, the consequences for patients and health care providers. Provide evidence for the model of BBCC and its effectiveness. Allow time for discussion / questions.
1.4	30	Understand the guiding style	Identify the key characteristics of the guiding style by contrasting two DVD clips of Ask students to identify the key characteristics of each style, record and compare on newsprint. BBCC – the one in a directing style and the other in a guiding style.
2.1	40	Reflective listening	Talk: Give a brief overview of the theory of reflective listening. Modelling: Demonstrate using DVD. Practice: Using small group interactive exercises.
2.2	40	Recognise, elicit and respond to change talk	Talk: Brief overview of theory from motivational interviewing. Practical: Trainers read out a list of statements and students drum on tables if they recognise change talk.
2.3	40	Introduction to the 5 As	Talk: Overview of the 5 A steps, the purpose of each step and communication skills involved. Allow time for discussion / questions.
3.1	80	Applying the 5 As to each risk factor	Form 4 groups: Each group looks at the training manual (5A steps and patient education material) for one behavioural risk factor. Form 4 new groups with one person from each of the previous groups. Each person teaches the others about their risk factor. Elicit feedback / discussion in whole group.
3.2	40	Exchanging information	Talk: Brief overview of theory from motivational interviewing. Modelling: Demonstrate elicit-provide-elicit with DVD. Practice: Small group interactive exercises.
4.1	30	Assess readiness to change	Talk: Brief overview of theory from motivational interviewing and application to the assess stage. Modelling: Demonstrate in role play or DVD. Practice: Small group interactive exercises.
4.2	60	Practice integrated BBCC	Groups of 4: Allocate one different risk factor per person. Each person thinks of a patient to role play. Role play BBCC. Observe, give feedback and discuss. Facilitator to rotate to each group.
4.3	25	Planning integration into real world	Interview each other in pairs: Assess how ready your partner is to implement BBCC. Assist the person appropriately to plan change. Each person briefly gives feedback on their way forward to whole group. Discuss ways of ongoing learning with group.
4.4	5	Closure and evaluation of workshop	Complete end of workshop with feedback form.

A checklist of all the educational resources required was developed. This included the PowerPoint slides needed for the talks, the DVD material, the instructions for the small group exercises, the course manual with information on risk factors, and the equipment needed. From this list the team decided which resources could be accessed from already available materials, and which resources needed to be developed.

The international Motivational Interviewing Network of Trainers (MINT) operates a website with extensive information about the clinical method and training of MI.^30^ This website has a manual containing a menu of exercises that MINT trainers use for various skills training. Practical exercises were chosen from the MINT manual, adapted for our setting, and used as practical exercises after a skill had been modelled.^30^

A training manual that summarised the model of BBCC and the underlying evidence, as well as applying the model practically to each risk factor, was printed for each participant. In addition each participant received, on each risk factor, patient educational material that was designed to dovetail with the approach to BBCC. All of these printed materials can be accessed via the web at www.ichange4health.co.za.

Both the researcher and Dr Katherine Everett-Murphy were trained internationally as MI trainers during the development of the course and became members of MINT.^30^ This experience was useful to improve their embodiment of the guiding style in the teaching approach, to update the practical exercises, and to incorporate the latest theory of MI.^13^

### Implementation

Twelve family medicine registrars, two general practitioners in private practice, and four family physicians were trained. Twenty three nurses on the one year Diploma course (Diploma in Clinical Nursing Science, Health Assessment, Treatment and Care) at the University of Stellenbosch were trained.

### Evaluation

Overall feedback on the conceptual model and content was positive. Doctors reported that the 5As framework was a useful structure, and that the patient information materials could help them save time during counselling. Nurses felt that reflective listening skills could help them feel less frustrated when counselling patients, as they conceptualised their potential roles as expert guides, rather than expert advice givers. Nurses found the course materials useful, not only as a source of information on NCDs, but also as resource when arranging for referral. Whilst participants found the interactive sessions (role-playing, group discussions and practical exercises) to be valuable in practicing new skills, they also identified the need for clearer instructions from trainers before exercises. Participants valued feedback from trainers during practical sessions, as they felt this improved their confidence in trying out new skills.

The training programme was adapted by the team using the participant's feedback, as well as their individual experiences during training. The team realised that, ideally, two trainers were needed during clinical skills training sessions to ensure individual feedback from a trainer. The need to encourage and strengthen the value of peer reviews was identified, therefore trainers purposefully selected group members to combine stronger and weaker members. To improve individual participation and save time, trainers provided clearer instructions before practical exercises.

## Discussion

This study has led to the design, development and implementation of a training programme for BBCC aimed at primary care providers (PCPs) in the South African context. The training programme incorporated best practice from both international and local studies and was tailored to the needs of local PCPs and their context. To our knowledge, this training programme is the first attempt at developing and implementing best practice BBCC training in our context, targeting a variety of PCPs, and addressing all four NCD risk factors.

The adaptation of the 5 A approach with a guiding style derived from MI was an innovation in the African context, and there are only a few other published examples internationally.^4,25,32,32^ Although approaches to training elsewhere include combinations of e-learning, with face-to-face training as well as ongoing support and feedback, they all aim to teach the underlying spirit of the guiding style of MI.^25,26,28,31, 32^ Training as part of continuing professional development through GP networks showed incomplete uptake, which could imply the need to integrate BBCC training into undergraduate and postgraduate programmes.^25^ One training programme which measured patient level outcomes was found ineffective, and interestingly did not train clinicians in listening skills, but focused more on the value of the guiding style, rather than achieving clinical competence.^32^

Training interventions for BBCC are not always clearly described and the terminology used is inconsistent.^33^ In an attempt to address this a review of BBCC and training resulted in a best practice checklist for training programmes.^26,28^ In line with the checklist our programme includes: clear information about the evidence base and theoretical underpinning of the model, tools for assessing readiness to change, reflective listening skills, provides tailored made information for patients, and topic specific training, whilst focussing on the development of essential communication skills by providing time for demonstrations, practice of skills and feedback. Our programme, however, does not include training on tailoring information specifically for different patient groups, such as the young, those with cultural differences, or minority ethnic groups.

The best practice checklist suggests that the individual context of participants, the trainer's attributes, as well as the process of delivery influence effectiveness. In our programme the participants were from a variety of linguistic, cultural, and professional contexts. In order to improve the trainer's understanding of their context, the programme always started with a session to elicit participant's prior experience, attitudes and expectations. Our trainers had completed training both in MI and in how to train MI, which enabled them to embody and evoke the guiding style during the training. We believe that trainers should have this level of proficiency, which may limit the immediate scaling up of training. If trainers with less proficiency are used then this may reduce the effectiveness of the training. Regarding the process of delivery, the workshop style used is supported by the checklist.^28^ However, ongoing support and feedback was not provided. Booster sessions to provide feedback and continuous support is regarded as important to maintain and develop skills over time.^28,34^ On-line support and feedback with the use of formative assessment of audiotapes is currently being developed to supplement this course.

The WIDER recommendations were developed to improve reporting of behaviour change interventions in clinical trials.^35^ This article is consistent with the WIDER recommendations in giving a detailed description of the intervention, which includes the characteristics of the trainers, the recipients, the setting, the mode of delivery, the intensity and duration of delivery, and a detailed description of the content. Likewise, the findings have described how the intervention was designed and developed, the techniques used to elicit change and the underlying conceptual model. A detailed manual has also been made available as a supplementary file. As this is not yet being assessed as part of a clinical trial the characteristics of a control group are not relevant here and the fidelity of the trainers to the design was not formally assessed.

A possible limitation of the ADDIE process is that there was less engagement with nurses, compared to doctors, in the design and development stages. For example, the design was presented mainly to expert doctors in primary care, and the educational team consisted of two family physicians and a social scientist. Nevertheless we did consult extensively with nurses during the situational analysis. During the implementation phase, the training was offered as an optional extra to nurses on the diploma programme as the time required was too much for the formal curriculum, and this could have selected a more motivated group. The majority of doctors were registrars in family medicine and their motivation and experience could also be different from other primary care doctors working in the public and private sector.

The study was conducted in the Western Cape, where the health system and human resources for health are generally better developed than elsewhere in the country. Additional contextual challenges might have been encountered if the programme had been developed in another province.

Evaluating the effect of training is good practice, and future research is focussing on evaluating the effectiveness of this training programme. The results of this evaluation will be published elsewhere. Evaluation should make it possible to determine the impact on clinical practice amongst the target groups, measure the degree to which the learning outcomes were met, and might also indicate which PCPs are best to train.^9,10,28^ Exploring the application of this programme to other PCPs, such as community health workers and lay counsellors, is also recommended for future research. Ultimately evaluation should measure the effect on patient behaviour and risk factors.

Although the training programme has not yet been fully evaluated, the need for it in our context has been immediately recognised and embraced. The Chronic Diseases Initiative for Africa, through a programme entitled ichange4health, helped to further develop the materials and train trainers from Departments of Family Medicine and Primary Care throughout South Africa. These trainers are now training medical students, general practitioners and other family physicians in their respective areas. A strength of this training intervention is its feasibility to train PCPs to train others in BBCC, and future research could evaluate the effectiveness of training the trainers. The authors have also presented the training programme in other countries such as Botswana and Namibia.

## Conclusion

This study designed a new approach to BBCC, which was based on a conceptual model that combined the 5As (ask, alert, assess, assist and arrange) with a guiding style derived from MI. The study then developed an eight-hour training programme that combined theory, modelling and simulated practice with feedback, delivered also in a guiding style, for either clinical nurse practitioners or primary care doctors in South Africa. The programme was implemented and revised based on initial feedback. The programme has already been widely adopted, but also requires further evaluation in our context.

## References

[1] Mayosi BM, Flisher AJ, Lalloo UG The burden of non-communicable diseases in South Africa. Lancet. 2009;374:934–947. http://dx.doi.org/10.1016/S0140-6736(09)61087-4 19709736 10.1016/S0140-6736(09)61087-4

[2] Bradshaw D, Steyn K, Levitt N, et al Non communicable diseases: A race against time [homepage on the Internet] Medical Research Council; 2011 Available from: http://www.mrc.ac.za/policybriefs/raceagainst.pdf.

[3] Goldstein MG, Whitlock E, De Pue MPH Multiple behaviour risk factor interventions in primary care. Summary of research evidence. Am J Prev Med. 2004;27:61–79. http://dx.doi.org/10.1016/j.amepre.2004.04.023 15275675 10.1016/j.amepre.2004.04.023

[4] Royal Australian College of General Practitioners. Smoking, nutrition, alcohol and physical inactivity (SNAP): A population health guide to behavioural risk factors in general practice [homepage on the Internet]. 2004 Available from: http://www.racgp.org.au/document.asp?id=?14803.

[5] Beaglehole R, Epping-Jordan J, Patel V Improving the prevention and management of chronic disease in low and middle income countries, priority for primary health care. Lancet 2008;372:940–949. http://dx.doi.org/10.1016/S0140-6736(08)61404-X 18790317 10.1016/S0140-6736(08)61404-X

[6] Department of Health, Republic of South Africa Strategic plan for the prevention and control of non-communicable diseases, 2012–2016.

[7] Spanou C, Simpson SA, Hood K Preventing disease through opportunistic, rapid engagement by primary care teams using behaviour change counseling (PRE-EMPT): Protocol for a general practice-based cluster randomized trial [serial on the Internet]. BMC Fam Pract. 2010 Available from: http://www.biomedcentral.com/1471-2296/11/69. 10.1186/1471-2296-11-69 PMC2955601 20858273

[8] Sim M, Wain T, Khong E Influencing behaviour change in general practice. Aust Fam Physician. 2009;38:885–888. 19893835

[9] Strayer S, Martindale J, Pelletier S Development and evaluation of an instrument for assessing brief behavioural change interventions. Patient Educ Couns. 2011;83:99–105. http://dx.doi.org/10.1016/j.pec.2010.04.012 20547030 10.1016/j.pec.2010.04.012

[10] Parker W, Steyn NP, Levitt NS They think they know but do they? Misalignment of perceptions of lifestyle modification knowledge among health professionals. Public Health Nutr. 2010;14:1429–1438. http://dx.doi.org/10.1017/S1368980009993272 20105391 10.1017/S1368980009993272

[11] Malan JE, Mash R, Everett-Murphy K A situational analysis of the current training and approaches to behaviour change counselling amongst primary health care nurses, doctors and key stakeholders in the Western Cape, South Africa. PHCFM Article. 2015.

[12] Everett–Murphy K, Odendaal H, Steyn K Doctor's attitudes and practices regarding smoking cessation during pregnancy. S Afr Med J. 2005;95:350–354. 15931451

[13] Miller WR, Rollnick S Motivational interviewing: Helping people change. 3rd ed New York, NY: Guildford Press, 2013; p. 392–393.

[14] Emmons K, Rollnick S Motivational interviewing in health care settings. Am J of Prev Med. 2001;20:68–74. http://dx.doi.org/10.1016/S0749-3797(00)00254-3 11137778 10.1016/s0749-3797(00)00254-3

[15] Allen WC Overview and evolution of the ADDIE Training System. Advances in Developing Human Resources [serial on the Internet]. 2006;8:430–441. Available from: http://adh.sagepub.com/content/8/4/430http://dx.doi.org/10.1177/1523422306292942

[16] Mash R Development of the programme Mental Disorders in Primary Care as internet-based distance education in South Africa. Med Educ [serial on the Internet]. 2001;35:996–999. http://dx.doi.org/10.1111/j.1365-2923.2001.01031. 10.1046/j.1365-2923.2001.01031.x 11564205

[17] Mash RM Diabetes education in primary care: A practical approach using the Addie model. CME. 2010;28:485–487.

[18] Mash R, Fairall L, Adejayan O, et al A morbidity survey of South African primary care. Plos One [serial on the Internet]. 2012;7(3):e32358 http://dx.doi.org/10.1371/journal.pone0032358. 10.1371/journal.pone.0032358 PMC3306367 22442666

[19] Mash R, Baldassini G, Mkhatshwa H Reflections on the training of counsellors in motivational interviewing for programmes for the prevention of mother to child transmission of HIV in sub-Saharan Africa. SA Fam Pract. 2008;50:53–59. http://dx.doi.org/10.1080/20786204.2008.10873697

[20] Everett-Murphy K, Steyn K, Matthews C, et al The effectiveness of adapted, best practice guidelines for smoking cessation counselling with pregnant smokers attending public sector antenatal clinics in Cape Town, South Africa. Acta Obstet et Gynecol Scand. 2010;89:478–490. http://dx.doi.org/10.3109/00016341003605701 10.3109/00016341003605701 20302533

[21] Malan Z The influence of information given on general practitioners’ management of overweight patients. Pretoria: University of Pretoria, Department of Family Medicine; 2008.

[22] Rollnick S, Butler CC, Kinnersly P, et al Competent novice motivational interviewing. Brit Med J. 2010;340:1242–1245. http://dx.doi.org/10.1136/bmj.c1900

[23] Lundahl B, Moleni T, Burke L, et al Motivational interviewing in medical settings: A systematic review and meta-analysis of randomised controlled trials. Patient Educ Couns. 2013;93:157–168. http://dx.doi.org/10.1016/j.pec.2013.07.012 24001658 10.1016/j.pec.2013.07.012

[24] Miller W, Mount KA A small study of training in MI: Does one workshop change clinician and client behaviour? Behav Cogn Psychother. 2001;29:457–471. http://dx.doi.org/10.1017/S1352465801004064

[25] Butler CC, Simpson SA, Hood K, et al Training practitioners to deliver opportunistic multiple behaviour change counselling in primary care: A cluster randomised trial. Brit Med J. 2013;346:1–25. http://dx.doi.org/10.1136/bmj.f1191 10.1136/bmj.f1191 PMC3601942 23512758

[26] Powell K, Thurston M Commissioning training for behaviour change interventions: Guidelines for best practice [document on the Internet]. 2008 Available from: http://hdl.handle.net//10034/46839.

[27] Torre DM, Daley BJ, Sebastian JL Overview of current learning theories for medical educators. Am J Med. 2006;119:903–907. http://dx.doi.org/10.1016/j.amjmed.2006.06.037 17000227 10.1016/j.amjmed.2006.06.037

[28] Stredder K, Sumnall H, Lyons M Behaviour change training delivered across Cheshire and Merseyside. A report mapping programmes and exploring processes [document on the Internet]. 2009 Available from: www.champs-for-health.net.

[29] Maguire P, Pitceathly P Clinical review: Key communication skills and how to acquire them. Brit Med J. 2002;325:697–700. http://dx.doi.org/10.1136/bmj.325.7366.697 12351365 10.1136/bmj.325.7366.697 PMC1124224

[30] Motivational Interviewing Network of Trainers (MINT) [document on the Internet] Available from: http://www.motivationalinterviewing.org

[31] Neuner-Jehle S, Schmid M, Gruninger U The health coaching programme: A new patient-centred and visually supported approach for health behaviour change in primary care [serial on the Internet]. BMC Fam Prac. 2013;14:1–8. Available from: http://www.biomedcentral.com/1471-2296/14/100 10.1186/1471-2296-14-100 PMC3750840 23865509

[32] Achhra A Health promotion in Australian general practice: A gap in GP training. Aus Fam Physician. 2009;38:605–608. 19893783

[33] Michie S, Van Stralen MM, West R The behaviour change wheel: A new method for characterising and designing behaviour change interventions [serial on the Internet]. Implement Sci. 2011;6:42 Available from: http://www.implementationscience.com/content/6/1/42.http://dx.doi.org/10.1186/1748-5908-6-42 21513547 10.1186/1748-5908-6-42 PMC3096582

[34] Schwalbe CS, Oh Hy, Zweben A Sustaining motivational interviewing: A meta-analysis of training studies. Addiction. 2014;109(8):1287–1294. http://dx.doi.org/10.1111/add.12558 24661345 10.1111/add.12558

[35] Albrecht L, Archibald M, Arseneau D, et al Development of a checklist to assess the quality of reporting of knowledge translation interventions using the Workgroup for Intervention Development and Evaluation Research (WIDER) recommendations. Implement Sci. 2013;8:52 http://dx.doi.org/10.1186/1748-5908-8-52 23680355 10.1186/1748-5908-8-52 PMC3661354

